# Intracellular Glutathione Depletion by Oridonin Leads to Apoptosis in Hepatic Stellate Cells

**DOI:** 10.3390/molecules19033327

**Published:** 2014-03-18

**Authors:** Liang-Mou Kuo, Chan-Yen Kuo, Chen-Yu Lin, Min-Fa Hung, Jiann-Jong Shen, Tsong-Long Hwang

**Affiliations:** 1 Graduate Institute of Clinical Medical Sciences, College of Medicine, Chang Gung University, Taoyuan 333, Taiwan; E-Mail: kuo33410@yahoo.com.tw; 2 Department of General Surgery, Chang Gung Memorial Hospital at Chia-Yi 613, Taiwan; 3 Graduate Institute of Natural Products, School of Traditional Chinese Medicine, Collage of Medicine, Chang Gung University, Taoyuan 333, Taiwan; E-Mails: cykuo@thu.edu.tw (C.-Y.K.); m9709108@stmail.cgu.edu.tw (C.-Y.L.); g902302@yahoo.com.tw (M.-F.H.); s0429@mail.cgu.edu.tw (J.-J.S.); 4 Chinese Herbal Medicine Research Team, Healthy Aging Research Center, Chang Gung University, Kweishan, Taoyuan 333, Taiwan

**Keywords:** apoptosis, glutathione, hepatic stellate cells, oridonin, reactive oxygen species

## Abstract

Proliferation of hepatic stellate cells (HSCs) plays a key role in the pathogenesis of liver fibrosis. Induction of HSC apoptosis by natural products is considered an effective strategy for treating liver fibrosis. Herein, the apoptotic effects of 7,20-epoxy-*ent*-kaurane (oridonin), a diterpenoid isolated from *Rabdosia rubescens*, and its underlying mechanisms were investigated in rat HSC cell line, HSC-T6. We found that oridonin inhibited cell viability of HSC-T6 in a concentration-dependent manner. Oridonin induced a reduction in mitochondrial membrane potential and increases in caspase 3 activation, subG1 phase, and DNA fragmentation. These apoptotic effects of oridonin were completely reversed by thiol antioxidants, *N*-acetylcysteine (NAC) and glutathione monoethyl ester. Moreover, oridonin increased production of reactive oxygen species (ROS), which was also inhibited by NAC. Significantly, oridonin reduced intracellular glutathione (GSH) level in a concentration- and time-dependent fashion. Additionally, oridonin induced phosphorylations of extracellular signal-regulated kinase (ERK), *c-Jun* N-terminal kinase (JNK), and p38 mitogen-activated protein kinase (MAPK). NAC prevented the activation of MAPKs in oridonin-induced cells. However, selective inhibitors of MAPKs failed to alter oridonin-induced cell death. In summary, these results demonstrate that induction of apoptosis in HSC-T6 by oridonin is associated with a decrease in cellular GSH level and increase in ROS production.

## 1. Introduction

Liver fibrosis is a common wound-healing response to various forms of chronic stimuli to the liver. The major cellular process of liver fibrosis is proliferation and activation of hepatic stellate cells (HSCs) [1,2,3]. During the pathogenesis of liver fibrosis, activated HSCs overproduce extracellular matrix proteins and pro-inflammatory growth factors [1,3]. Increasing evidence indicates that decrease in number of activated HSCs by inhibiting proliferation or induction of apoptosis in HSCs can be a useful therapeutic strategy for liver fibrosis [4]. Herbal natural products exhibit potential roles in preventing or treating liver fibrosis [5]. Therefore, studying the action mechanisms of potential natural compounds is important in developing better remedial strategies for liver disorders.

**Figure 1:**
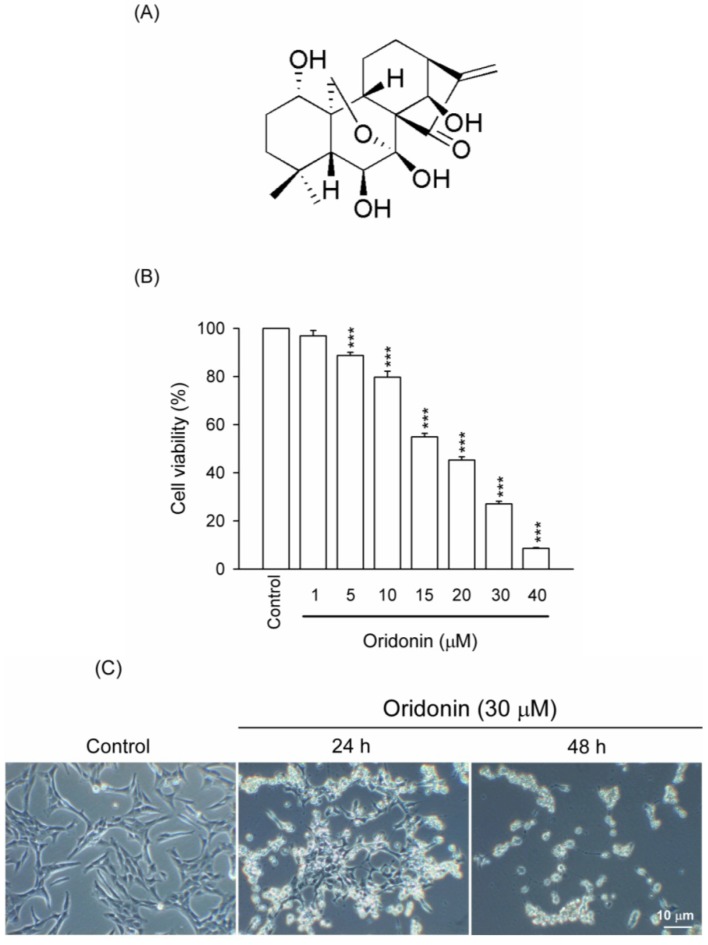
Oridonin decreased viability of HSC-T6. Cells were treated with DMSO (control) or indicated concentrations of oridonin for 24 h. (**A**) Chemical structure of 7,20-epoxy-ent-kaurane (oridonin). (**B**) After the incubation period, cell viability was determined using WST-1 assay. (**C**) Morphological changes in HSC-T6 were observed at 0 (control), 24, and 48 h. Bar = 10 μM. All data are presented as the mean ± SEM. (*n* = 6). *** *p* < 0.001 compared to control.

Donglingcao (*Rabdosia rubescens*) has been used as a traditional medicine for the treatment of various cancers and inflammatory disorders [6,7]. Diterpenoids are the major active constituents of *Rabdosia rubescens* [8,9]. 7,20-Epoxy-*ent*-kaurane (oridonin, Figure 1A), a diterpenoid extracted from *Rabdosia rubescens*, has various pharmacological and biological effects, such as anti-inflammatory, antiviral, and antibacterial activities [10,11]. Recently, many studies have suggested that oridonin has potent anticancer activities against a number of cancers, such as lung cancer, colorectal cancer, and hepatocarcinoma, *in vitro* and *in vivo* [6,12,13,14]. Liver fibrosis is well known to increase the risk of developing hepatocarcinoma [15,16]. However, the pharmacological effects of oridonin and its underlying mechanisms in HSCs are still unknown.

The aim of this study was to investigate the apoptotic effects of oridonin in rat HSC cell line (HSC-T6) and its action mechanisms. The intracellular signaling pathways responsible for proliferation and activation of HSCs are complex and need to be further studied [4]. Intracelluar glutathione (GSH) level has a considerable responsibility to maintain intracellular redox homeostasis and cell viability in HSCs [17]. Experimental evidences indicate that reactive oxygen species (ROS) accumulation caused by GSH depletion can induce caspase 3 activation and cell apoptosis [18,19]. For that reason, it is important to identify candidate compounds that induce HSC apoptosis and prevent the progression of hepatic fibrosis through depletion of GSH and overproduction of ROS. Our results show that oridonin significantly induces apoptosis by decrease in intracellular GSH concentration in HSC-T6.

## 2. Results and Discussion

### 2.1. Oridonin Inhibited Cell Viability and Induced Apoptosis in HSC-T6 Cells

**Figure 2:**
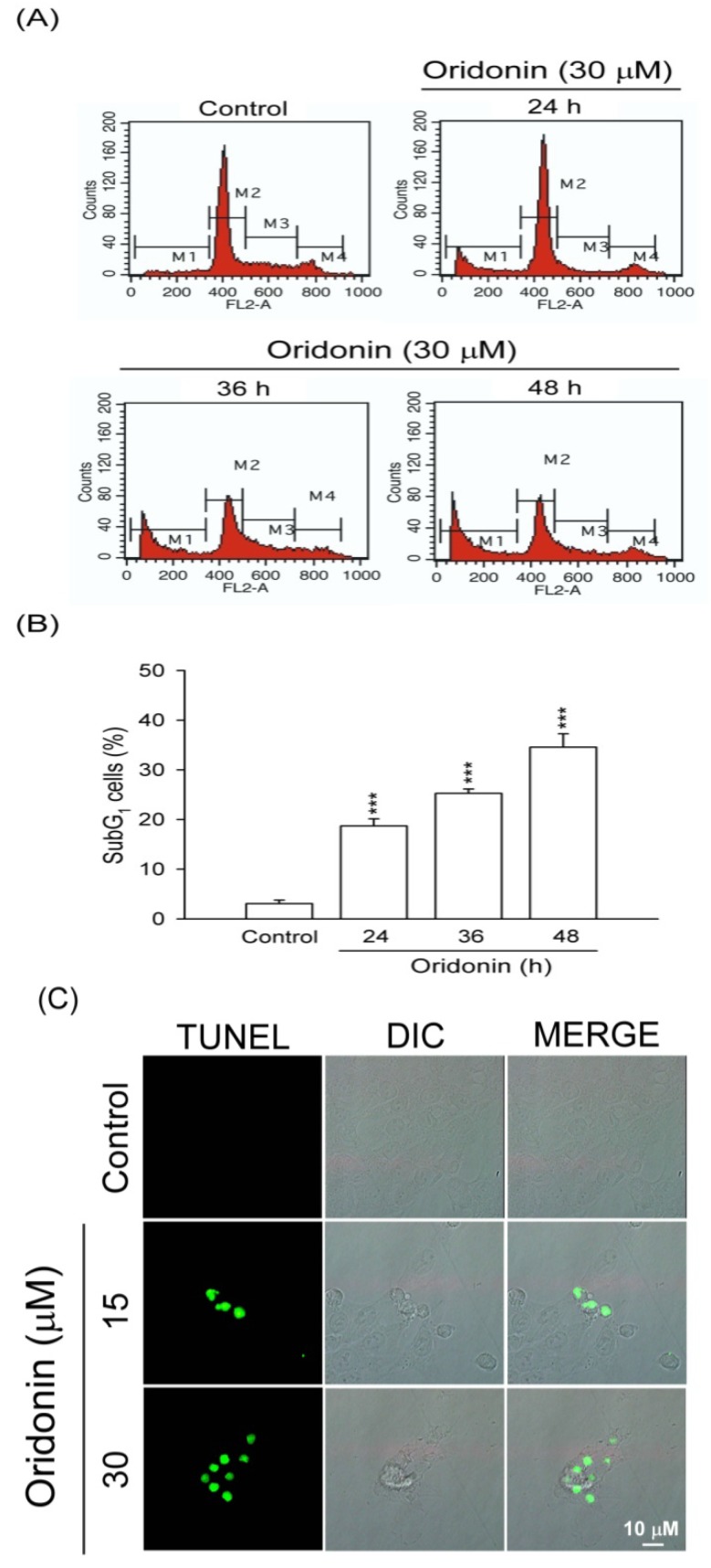
Oridonin induced apoptosis of HSC-T6. (**A**) Time-dependent changes in the subG1 phase population were determined after oridonin (30 μM) treatment or not (control). (**B**) Representative subG1 populations calculated from FACS histograms are shown (*n* = 4). (**C**) Changes in nuclear morphology by DMSO (control) or oridonin at 24 h were visualized using TUNEL staining. Bar = 10 μM. All data are presented as the mean ± SEM. *** *p* < 0.001 compared to control.

Oridonin (5–40 μM) inhibited cell viability of HSC-T6 in a concentration-dependent manner with an IC_50_ value of 16.94 ± 0.47 μM (Figure 1B). To characterize the oridonin-induced cell death of HSC-T6, we observed the changes in cellular morphology. Phase-contrast microscopy showed that oridonin (30 μM) treated cells for 24 and 48 h underwent marked apoptotic changes, including formation of membrane blebs and apoptotic bodies (Figure 1C). To further determine the apoptotic features, cell cycle and TUNEL staining were assayed. Oridonin (30 μM) caused an increase in subG1 phase, which is an indicator of apoptosis, in a time-dependent fashion (Figure 2A,B). In addition, DNA fragmentation after treatment with oridonin (15 and 30 μM) was observed (Figure 2C).

**Figure 3:**
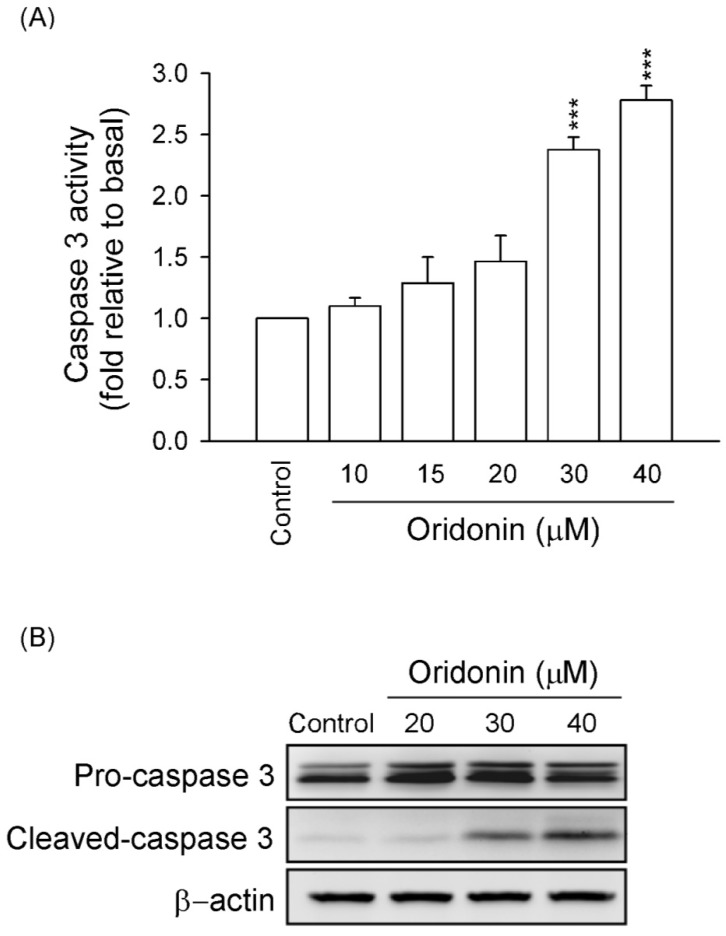
Oridonin increased caspase 3 activity and expression. HSC-T6 cells were treated with different concentrations of oridonin for 24 h. (**A**) Caspase 3 activity was measured using Caspase 3/CPP32 colorimetric assay kits. (*n* = 6). *** *p* < 0.001 compared to control. (**B**) The expressions of pro-caspase and cleaved caspase 3 were detected using western blotting analysis. β-Actin was used as a loading control. All data are presented as the mean ± SEM.

It is well known that caspase 3 has a central role in the apoptotic responses. Our results showed that activity of caspase 3 and expression of cleaved caspase 3 were significantly increased in oridonin (30 and 40 μM)-treated HSC-T6 (Figure 3). These results suggested that oridonin induced apoptosis of HSC-T6 through caspase 3-dependent pathway.

### 2.2. Oridonin Induced Intracellular ROS Generation

**Figure 4:**
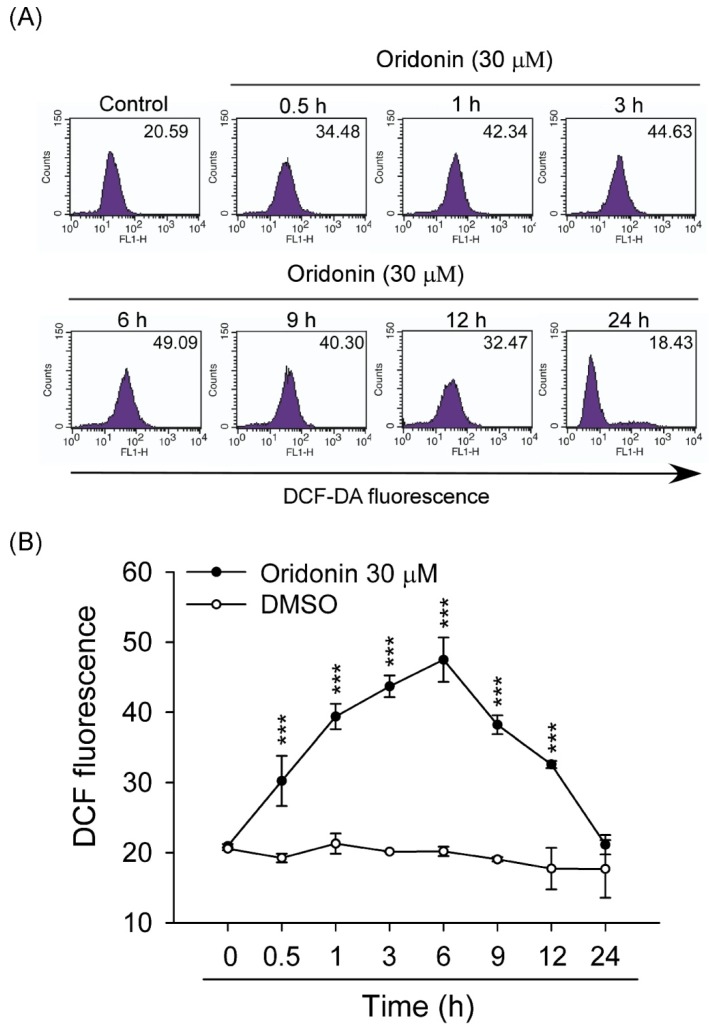
Oridonin induced ROS production. HSC-T6 cells were treated with oridonin for the indicated times. The levels of intracellular ROS were determined using DCF-DA, and the fluorescence was detected using FACS Calibur analysis. (**A**) Right shifts in fluorescence represented a change induced by oridonin (30 μM) or not (0 h, control). (**B**) The mean fluorescence intensity of DMSO (control) or oridonin is shown. (*n* = 6–8). All data are presented as the mean ± SEM. *** *p* < 0.001 compared to control.

**Figure 5:**
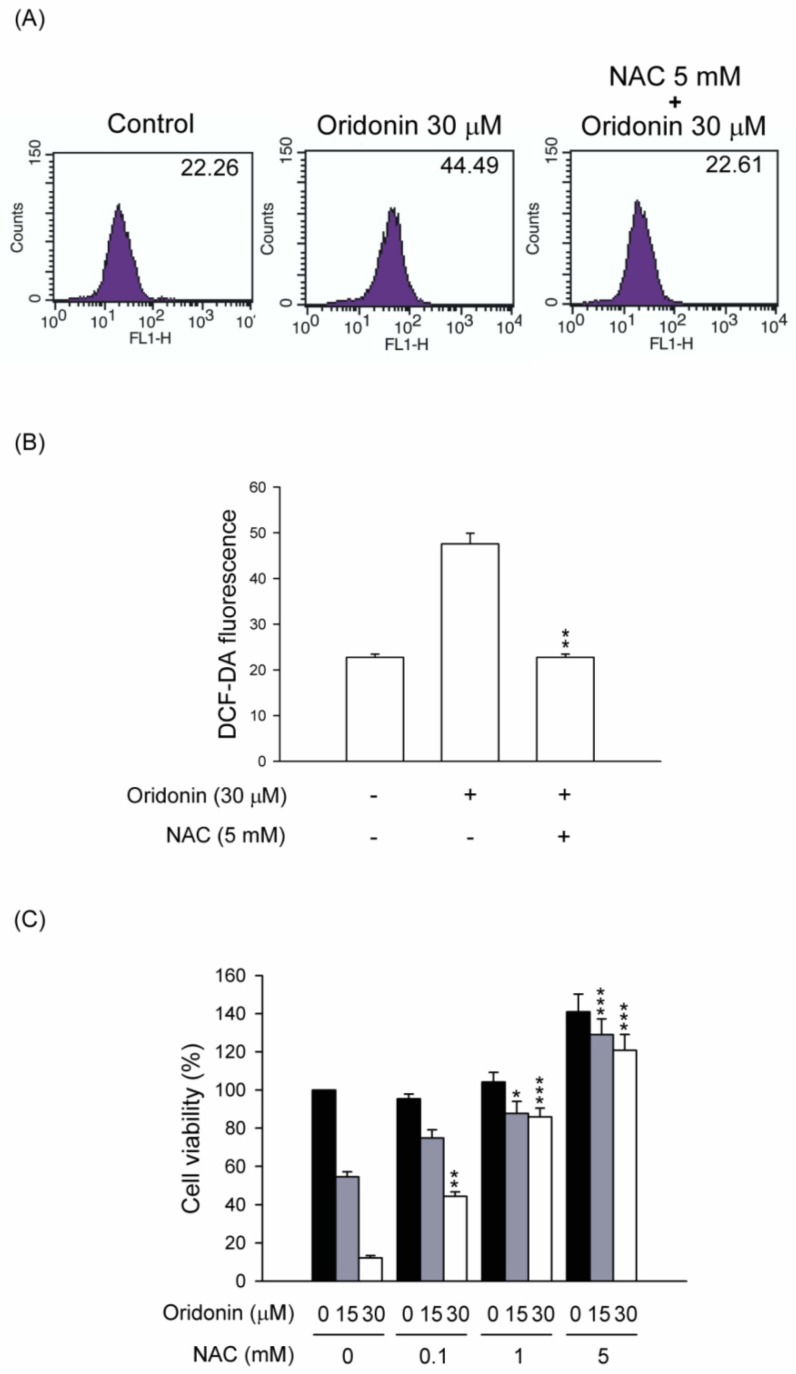
NAC inhibited ROS production and cell viability in oridonin-treated HSC-T6. (**A**) Cells were pretreated with NAC (5 mM) for 1 h and then treated with DMSO (control) or oridonin (30 μM) for 6 h. The levels of intracellular ROS were determined using DCF-DA, and the fluorescence was detected using FACS Calibur analysis. (**B**) The mean fluorescence intensity is shown. (**C**) NAC reversed the oridonin-induced cell death. NAC (0.1, 1, and 5 mM) was preincubated for 1 h before the addition of oridonin (15 and 30 μM) for 24 h. Cell viability was detected using the WST-1 assay (*n* = 4). All data are presented as the mean ± SEM. * *p* < 0.05; ** *p* < 0.01; *** *p* < 0.001 compared to the corresponding oridonin alone.

To study the effect of oridonin on oxidative stress, intracellular ROS generation was determined. We found that oridonin (30 μM) significantly induced ROS generation within 24 h and has a peak effect at 6 h (Figure 4). NAC, a thiol-antioxidant, inhibited the oridonin-induced ROS generation in HSC-T6 cells (Figure 5A,B). Furthermore, NAC (0.1, 1, and 5 mM) significantly reduced oridonin (15 and 30 μM)-caused cell death (Figure 5C).

### 2.3. Oridonin Reduced Intracellular GSH Level

**Figure 6:**
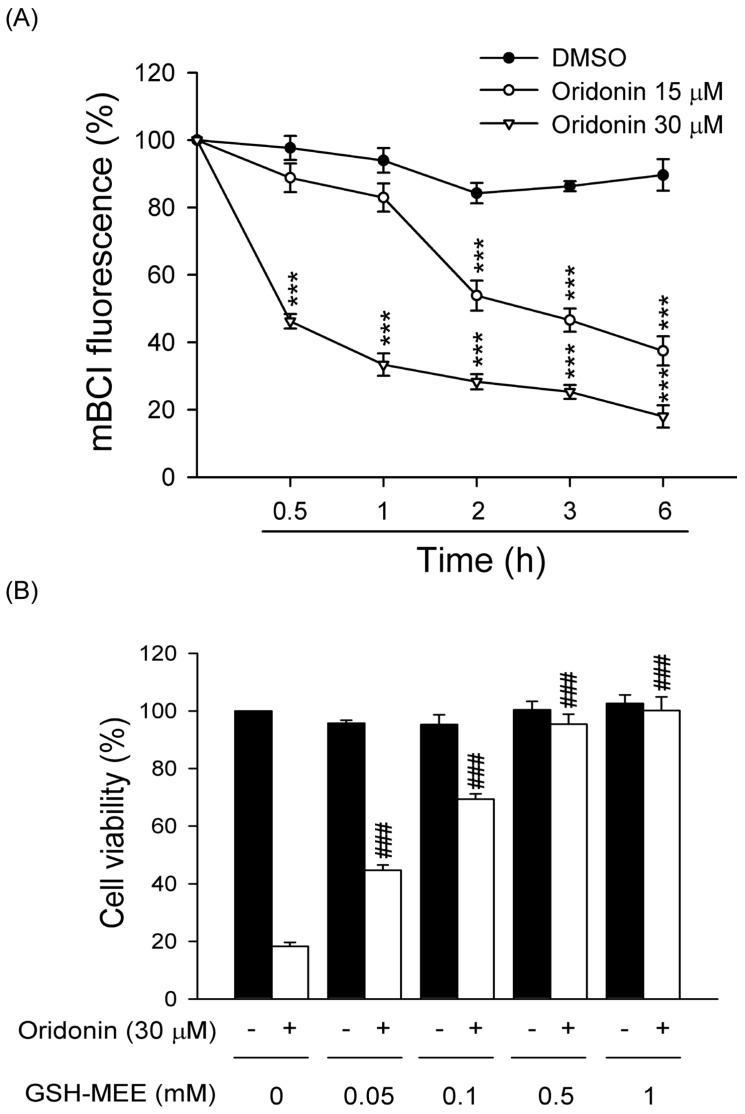
Oridonin caused intracellular GSH depletion. (**A**) Cells were treated with DMSO or oridonin (15 or 30 μM) for the indicate times. Intracellular GSH levels were determined using mBCl. (*n* = 6). (**B**) GSH-MEE reversed the cell death caused by oridonin. GSH-MEE (0.05, 0.1, 0.5, and 1 mM) was preincubated for 1 h before the addition of oridonin (30 μM) for 24 h. Cell viability was detected using the WST-1 assay (*n* = 4). All data are presented as the mean ± SEM. *** *p* < 0.001 compared to DMSO; ^###^ *p* < 0.001 compared to oridonin alone.

GSH concentration has an important effect on intracellular redox homeostasis. To investigate whether oridonin reduces intracellular GSH level in HSC-T6 cells, the amount of intracellular GSH was assayed. Oridonin (15 and 30 μM) significant reduced GSH level in a concentration- and time-dependent manner (Figure 6A). Furthermore, to further confirm the oridonin-induced cell death is mediated through GSH depletion, the effect of GSH-MEE, a membrane permeable derivative of GSH, on oridonin-induced cell viability was tested. Our results showed that GSH-MEE (0.05–1 mM) significantly reduced oridonin-caused cell death in a concentration-dependent manner (Figure 6B).

### 2.4. Effect of Oridonin on Phosphorylation of MAPKs

**Figure 7:**
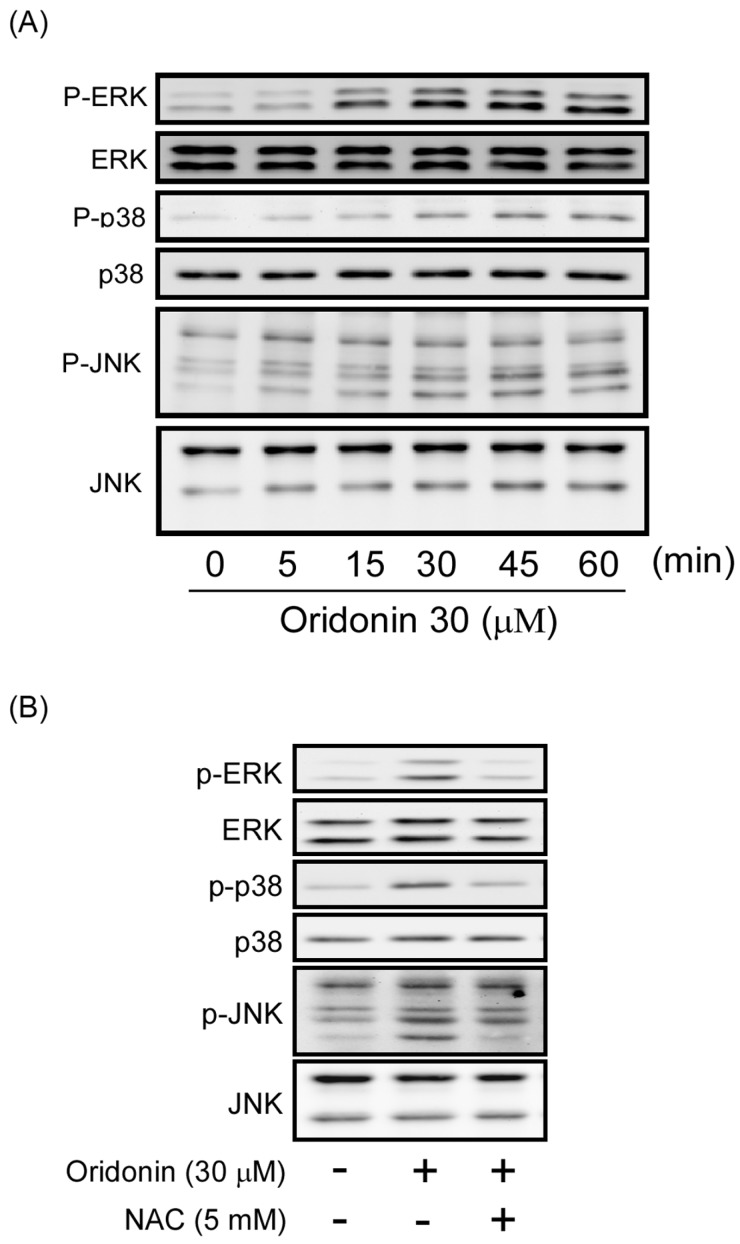
Oridonin induced phosphorylation of MAPKs. (**A**) Oridonin induced the phosphorylation of MAPKs in a time-dependent manner. Cells were treated with oridonin (30 μM) for the indicated times. Phosphorylation of ERK, p38 MAPK, and JNK was analyzed using immunoblotting analysis with antibodies against phosphorylated and total protein. (**B**) NAC reversed oridonin-induced phosphorylation of MAPKs. NAC (5 mM) was preincubated for 1 h before the addition of oridonin (30 μM). Phosphorylation of ERK, p38 MAPK, and JNK was analyzed using immunoblotting analysis with antibodies against the phosphorylated and total protein.

**Figure 8:**
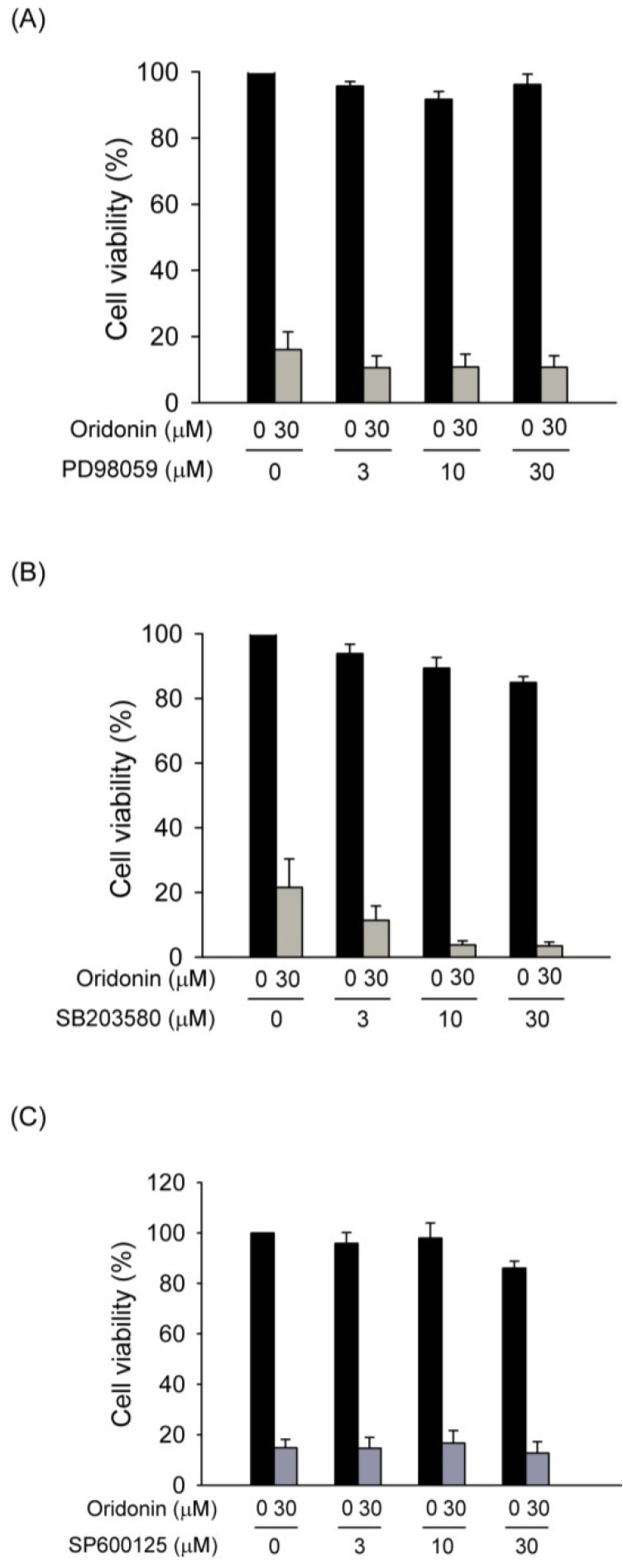
Pharmacological inhibitors of MAPKs failed to alter oridonin-induced cell death. (**A**) PD98059, (**B**) SB203580, and (**C**) SP600125 did not reverse the cell death caused by oridonin. Cells were pretreated with various concentrations of inhibitors for 1 h before the addition of oridonin (30 μM). Cell viability was detected using the WST-1 assay (*n* = 3).

MAPK pathway is known to have a significant role in cell growth and/or apoptosis. To examine whether MAPKs is involved in the oridonin-induced apoptosis of HSC-T6, activation of these kinases was assayed by Western blotting. Oridonin (30 μM) induced rapid phosphorylation of ERK, p38 MAPK, and JNK (Figure 7A). Phosphorylation of ERK, p38 MAPK, and JNK by oridonin was reversed by NAC (5 mM) (Figure 7B). However, the specific inhibitors of p38 MAPK (SB203580), JNK (SP600125), and ERK (PD98059) failed to alter oridonin-induced apoptosis (Figure 8).

### 2.5. NAC Inhibited the Changes of Mitochondrial Membrane Potential, Caspase 3 Activity, and Subg1 Population in Oridonin-Treated HSC-T6 Cells

**Figure 9:**
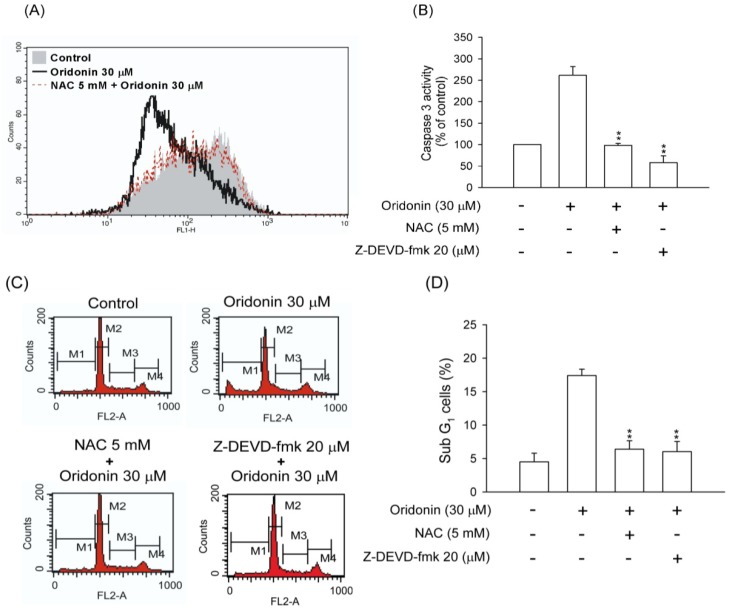
NAC and Z-DEVD-fmk reversed oridonin-induced mitochondrial dysfunction, caspase 3 activation, and DNA fragmentation. (**A**) NAC reversed the oridonin-induced loss of mitochondrial membrane potential. NAC (5 mM) was preincubated for 1 h before the addition of oridonin (30 μM) for 24 h. The mitochondrial membrane potential was determined using rhodamine 123, and the fluorescence was detected using FACS Calibur analysis. The mean fluorescence intensity is shown in the upper right corner (*n* = 4). (**B**) NAC and Z-DEVD-fmk reversed oridonin-induced caspase 3 activity. NAC (5 mM) or Z-DEVD-fmk (20 μM) was preincubated for 1 h before the addition of oridonin (30 μM) for 24 h. The caspase 3 activity was determined using Caspase 3/CPP32 colorimetric assay kits. (**C**) NAC and Z-DEVD-fmk suppressed oridonin-induced DNA fragmentation. NAC (5 mM) or Z-DEVD-fmk (20 μM) was preincubated for 1 h before the addition of oridonin (30 μM) for 24 h. Changes in the subG1 phase ratio were determined using propidium iodide (PI) staining at the indicated time points using FACS Calibur analysis. (**D**) Representative subG1 populations calculated from FACSalibur histograms are shown (*n* = 6). All data were presented as the mean ± SEM. ** *p* < 0.01 compared to oridonin alone.

Accumulation of ROS may result in a decrease in the mitochondrial membrane potential and trigger cell apoptosis [20,21]. Oridonin decreased the mitochondrial membrane potential, and pretreatment with NAC reversed this effect (Figure 9A). Furthermore, to determine whether ROS contributed to apoptosis in a caspase 3 dependent manner, the effects of NAC and specific caspase 3 inhibitor, Z-DEVD-fmk, were tested. Our results found that NAC and Z-DEVD-fmk not only suppressed caspase 3 activity (Figure 9B) but also inhibited subG1 population in oridonin-induced cells (Figure 9C,D).

### 2.6. Discussion

Activated HSCs play a central role in the pathogenesis of liver fibrosis [1,2,22]. For that reason, stimulation of HSC apoptosis by herbal natural compounds has been considered an attractive therapeutic strategy for treatment and/or prevention of liver fibrosis [1,23,24]. However, there are currently not clinical drugs that directly modulate HSC activation and proliferation. Many reports suggest that oridonin, a natural diterpenoid isolated from *Rabdosia rubescens*, has potent anti-hepatocarcinoma activities *in vitro* and *in vivo* [13,20,25,26]. It is well-known that liver fibrosis can lead to liver cancers [15,16,26]. However, the pharmacological effects of oridonin in HSCs are still unknown. In this study, our data show that oridonin significantly and concentration-dependently induces cell apoptosis in HSCs.

Several lines of evidences support the potential importance of inducing apoptosis of HSC for the regression of liver fibrosis [1,22,27,28,29]. Oridonin had a significant inhibitory effect on the viability of HSCs. We found that oridonin caused noticeable apoptotic changes, including formation of membrane blebs and apoptotic bodies. To confirm apoptotic effects, we further studied the mitochondrial membrane potential, DNA fragmentation and caspase activity. Oridonin induced significant increases in SubG1 population, DNA fragmentation, and caspase 3 expression and activity, and it caused a reduction in mitochondrial membrane potential. These apoptotic effects of oridonin were completely reversed by Z-DEVD-fmk, a specific caspase 3 inhibitor. Activation of caspase 3 is a fundamental role in the apoptotic responses. These results suggest that oridonin stimulates apoptosis in HSC-T6 through caspase 3-dependent pathway. Our results are in keeping with those reported by Bohanon *et al.* [30] who showed that oridonin not only induced cell apoptosis but also reduced expression of α-SMA, type I collagen, and fibronectin in LX-2 cells, an activated human HSCs. Together, these findings demonstrate that oridonin may have potential to treat liver fibrosis.

GSH is a main intracellular antioxidant and plays an important role in the regulation of cell viability in HSCs [17]. GSH has an inhibitory effect against ROS-induced apoptotic cell death [19]. Therefore, induction of apoptosis in HSCs by decrease in cellular GSH concentration may provide a novel approach for treating liver fibrosis. We showed that oridonin significantly decreased intracellular GSH concentration and led to apoptosis in HSCs. Intracellular GSH level is increased in the presence of the cell-permeable derivative GSH-MEE, which is hydrolyzed by esterases [18]. GSH-MEE significantly restored cell viability caused by oridonin. These results support our hypothesis that oridonin enhances ROS production and apoptosis in HSCs by decrease in cellular GSH concentration. Moreover, NAC, a GSH precursor, reversed oridonin-induced ROS accumulation and apoptosis. Consistent with previous reports, GSH depletion increases the sensitivity of HSCs to oxidative stress-induced cell death [19,31]. In contrast, oridonin was shown to protect oxidative stress-induced apoptosis in keratinocytes and epidermoid carcinoma cells [32,33]. Experimental evidence showed that ROS have a pathological effect in many types of liver diseases [34,35,36]. The controversial results may be due to different cell types. Clearly, the precise roles of ROS in liver as a regulatory, protective, or deleterious mediator are still unresolved questions and need to be further investigated.

A recent report showed that oridonin causes apoptotic effects in metastatic hepatocellular carcinoma cells through a mitochondrial pathway [37]. In support of this mechanism, mitochondrial membrane potential was decreased by the treatment of oridonin in HSCs. Mitochondria is an important source of intracellular ROS [38]. However, the mitochondrial complex I inhibitor, rotenone, did not reduce the oridonin-induced ROS formation and cell apoptosis (data not shown). Because the oridonin-caused decrease of mitochondrial membrane potential was reversed by NAC, we suggest that accumulation of ROS by oridonin leads to reduction in mitochondrial membrane potential and trigger cell apoptosis. 

MAPKs, such as ERK, JNK, and p38 MAPK, are important mediators of signal transduction in oxidative stress-elicited cell responses [39,40]. There have been reported that MAPK signaling pathway is involved in cell growth and activation in HSCs [20,41]. Previous report showed that oridonin-induced apoptosis of HepG2 is through ROS induced MAPK activation [20]. In contrast, Yu and colleagues found that continuously generated H_2_O_2_ caused inhibition of cell growth of human gingival fibroblasts is independent of MAPK activation [42]. Noticeably, MAPK pathway in the oxidative stress-induced apoptosis of HSCs is still uncertain. Oridonin significantly induced phosphorylation of MAPKs in HSCs. However, pharmacological inhibitors of p38 MAPK (SB203580), JNK (SP600125), and ERK (PD98059) failed to alter oridonin-induced apoptosis in HSCs. Therefore, we speculated that the apoptotic effects of oridonin in HSCs were not mediated by activation of p38 MAPK, JNK, and ERK. Although the role of MAPK activation in oridonin-mediated effects in HSCs is still unknown, our data showed that NAC completely inhibited oridonin-stimulated activation of p38 MAPK, JNK, and ERK. The results indicate that activation of MAPKs by oridonin in HSCs is mediated by depletion of GSH.

## 3. Experimental

### 3.1. Reagents

Oridonin and glutathione monoethyl ester (GSH-MEE) were purchased from Calbiochem (La Jolla, CA, USA). Z-DEVD-fmk and the Caspase 3/CPP32 colorimetric assay kit were obtained from BioVision (Mountain, PA, USA). The cell proliferation reagent WST-1 and RNase A were obtained from Roche Applied Sciences (Mannheim, Germany). Antibodies against phospho-ERK1/2, ERK1/2, phospho-JNK, and JNK were purchased from Cell Signaling (Beverly, MA, USA). Phospho-p38 and p38 MAPK were obtained from Santa Cruz Biotechnology (Santa Cruz, CA, USA). Other chemicals were purchased from Sigma (St. Louis, MO, USA).

### 3.2. Cell Culture

HSC-T6, a rat HSC cell line, was kindly provided by Professor Scott L. Friedman (Mount Sinai School of Medicine, NY, USA). HSC-T6 cells were cultured at 37 °C in Dulbecco’s minimum essential medium (DMEM; Gibco, Grand Island, NY, USA) supplemented with 10% fetal bovine serum (FBS) and antibiotics (100 U/mL penicillin, 100 μg/mL streptomycin, and 2.5 μg/mL amphotericin B) in a humidified atmosphere with 5% CO_2_. The culture medium was replaced every other day. Once the cells reached 70%–80% confluency, they were trypsinized and seeded onto 6- or 24-well plastic dishes for further experiments.

### 3.3. Cell Viability Assay

Cell viability was measured using WST-1 assay. HSC-T6 cells were seeded at a density of 5 × 10^4^ cells/mL in 24-well plates and cultured in phenol red-free DMEM containing 0.5% heat-inactivated FBS for 24 h. After incubation, cells were incubated with indicated concentrations of oridonin for 24 h. The WST-1 reagent was then added into the medium and incubated at 37 °C for 2 h. The absorbance was measured at 450 nm in a microplate reader (Thermo Labsystems, Waltham, MA, USA).

### 3.4. Analysis of Cell Cycle

Cells were treated with oridonin at the indicated times before harvesting and fixing in ice-cold 70% ethanol for 1 h. Cells were then washed with PBS and incubated in propidium iodide staining buffer (50 μg/mL propidium iodide, 0.1 mg/mL DNase-free RNase A, and 0.5% Triton X-100) for 30 min at 37 °C in the dark. The DNA content was analyzed by flow cytometry.

### 3.5. Measurement of Intracellular ROS Generation

The measurement of intracellular ROS generation was performed using 2',7'-dichlorofluorescin diacetate (DCF-DA; Sigma) reagent. The relative level of ROS was measured by flow cytometry (BD Biosciences, San Jose, CA, USA).

### 3.6. Measurement of Intracellular GSH Levels

Intracellular GSH levels were assayed by fluorescent monochlorobimane (mBCl; Molecular Probes, Eugene, OR, USA) according to the manufacturer’s instructions. Briefly, after treatment of cells with 30 μM oridonin, the cells were re-incubated in DMEM containing 100 μM mBCl at 37 °C for 30 min in the dark. Fluorimetric analysis was performed using a fluorescence plate reader with 400 nm excitation/505 nm emission filters (Fusion™, Packard BioScience, Waltham, MA, USA).

### 3.7. Caspase 3 Activity Assay

The activity of caspase 3 was assayed using a Caspase 3/CPP32 colorimetric assay kit. Cells were treated with oridonin for 2 h, and then resuspended in cell lysis buffer (BioVision, Milpitas, CA, USA). Samples were centrifuged at 10,000 *g* for 5 min at 4 °C to yield cell lysates. Protein concentrations of cell lysates were determined by the Bradford assay using bovine serum albumin (BSA) as a standard. The isolated supernatant was mixed with an equal volume of reaction buffer (containing 10 mM DTT and 400 μM DEVD-*p*NA) and incubated at 37 °C for 120 min. The absorbance was measured at 450 nm in a microplate reader (Thermo Labsystems).

### 3.8. Western Blotting

Cells were pelleted and resuspended in ice-cold relaxation buffer (20 mM Tris-HCl (pH 7.4), 150 mM NaCl, 1 mM EGTA, 1 mM NaF, 2 mM Na_3_VO_4_, 1 mM phenylmethylsulfonyl fluoride, 1% dilution of Sigma protease cocktail, and 1% Triton X-100). Samples were centrifuged at 14,000 *g* for 20 min at 4 °C to yield cell lysates. Proteins were separated by 10% or 12% sodium dodecyl sulfate-polyacrylamide gel electrophoresis (SDS-PAGE) and electrophoresed onto a nitrocellulose membrane. Immunoblotting was performed using specific primary antibodies and horseradish peroxidase-conjugated secondary antibodies (Cell Signaling), and peroxidase activity was assessed using an enhanced chemiluminescence kit (Perkin-Elmer Life Science, Boston, MA, USA). The intensities of the reactive bands were analyzed using UVP Biospectrum (UVP LLC, Upland, CA, USA).

### 3.9. Assay of Mitochondrial Membrane Potential

Rhodamine-123 is used as a probe to detect the mitochondrial membrane potential. Cells were treated with oridonin for 24 h, and then incubated with 1 μg/mL of rhodamine 123 at 37 °C for 1 h in the dark. The fluorescence intensity of Rhodamine 123 was measured by flow cytometry.

### 3.10. Apoptosis Staining

Apoptosis-associated DNA fragmentation was visualized using the terminal deoxyribonucleotidyl transferase-mediated dUTP-digoxigenin nick end labeling (TUNEL) apoptosis detection kit (Roche, Mannheim, Germany). The cells were finally counterstained with DAPI and analysed using an OLYMPUS IX 81 microscope (Olympus, Tokyo, Japan).

### 3.11. Statistical Analysis

Data were expressed as the mean ± standard error of mean (SEM), and comparisons were statistically calculated by one-way or two-way analysis of variance (ANOVA) followed by Bonferroni *post-hoc* test. A value of *p* < 0.05 was considered statistically significant.

## 4. Conclusions

In the present study, we show that oridonin, a bioactive diterpenoid isolated from *Rabdosia rubescens*, induces apoptosis in HSCs. Multiple observations made in the study indicate that the apoptotic effects of oridonin in HSCs are through decrease of intracellular GSH concentration and increase of ROS formation and caspase activation. An apoptotic action in activated HSCs may be used to control liver fibrosis. Our pharmacological findings support further development of oridonin as a novel therapeutic agent for treating HSC-related liver fibrosis. 
